# Synergistic Beneficial Effects of Resveratrol and Diet on High-Fat Diet-Induced Obesity

**DOI:** 10.3390/medicina58091301

**Published:** 2022-09-18

**Authors:** Osama Abo Alrob, Ramzi A. Al-Horani, Zaid Altaany, Mohammad B. Nusair

**Affiliations:** 1 Clinical Pharmacy and Pharmacy Practice Department, Faculty of Pharmacy, Yarmouk University, Irbid 211-63, Jordan or; 2 Department of Exercise Science, Yarmouk University, Irbid 211-63, Jordan; 3 Department of Basic Sciences, Faculty of Medicine, Yarmouk University, Irbid 211-63, Jordan; 4 Department of Sociobehavioral and Administrative Pharmacy, College of Pharmacy, Nova Southeastern University, Fort Lauderdale, FL 33314, USA

**Keywords:** resveratrol, obesity, insulin sensitivity, fatty acid oxidation

## Abstract

*Introduction*: Despite decades of research, obesity and its related medical complications remain a major health concern globally. Therefore, novel therapeutic strategies are needed to combat obesity and its numerous debilitating complications. Resveratrol (RES) has a potential therapeutic effect in obesity and diabetes by improving oxidative metabolism and insulin signaling. *Background and Objectives*: The aim of this study was to investigate the effect of RES treatment on weight loss and glucose and fatty acid metabolism. Methods: Obesity was induced in 24 mice by exposure to a high-fat diet (HFD) for 8 weeks. Mice were randomly assigned to one group of either: group 1: control, non-treated low-fat diet (LFD) for 12 weeks (*n* = 8), group 2: non-treated high-fat diet (HFD) for 12 weeks (*n* = 8), group 3: RES-treated HFD (HFD + RES) (*n* = 8), or group 4: RES-treated and switched to LFD (HFD-LFD + RES) (*n* = 8). HFD + RES mice were first fed an HFD for 8 weeks followed by 4 weeks of RES. The HFD-LFD + RES group was first fed an HFD for 8 weeks and then treated with RES and switched to an LFD for 4 weeks. *Results*: After 12 weeks, group 2 mice had significantly higher body weights compared to group 1 (23.71 ± 1.95 vs. 47.83 ± 2.27; *p* < 0.05). Group 4 had a significant decrease in body weight and improvement in glucose tolerance compared to mice in group 2 (71.3 ± 1.17 vs. 46.1 ± 1.82 and 40.9 ± 1.75, respectively; *p* < 0.05). Skeletal muscles expression of SIRT1, SIRT3, and PGC1α were induced in group 3 and 4 mice compared to group 2 (*p* < 0.01), with no changes in AMP-activated protein kinase expression levels. Furthermore, combination of RES and diet ameliorated skeletal muscle intermediate lipid accumulation and significantly improved insulin sensitivity and secretion. *Conclusions*: The results of this study suggest a synergistic beneficial effect of LFD and RES to lower body weight and enhance glucose and fatty acid metabolism.

## 1. Introduction

Obesity is a complex multifactorial syndrome associated with extensive accumulation of visceral fat that poses a risk to health. The prevalence of obesity has increased worldwide among both genders [1,2]. In addition, obesity’s complications contribute significantly to increased incidences of mortality and morbidity [3,4]. Current treatment regimens of obesity include dietary and lifestyle interventions, bariatric surgeries, and few pharmacological drugs such as glucagon-like peptide-1 (GLP-1) agonists [5,6]. Classical pharmacological and non-pharmacological treatment strategies can improve insulin sensitivity and enhance survival chances in obese patients. However, these strategies are inadequate in curing the insulin insensitivity seen in obesity and type 2 diabetes or preventing disease progression [7]. The lack of an effective pharmaceutical treatment has urged substantial investment in the research and development of natural products that offer broad pharmacological activities acting via multiple mechanisms on pathophysiological signaling in obesity. Resveratrol (RES) is a natural polyphenolic compound that is found in plant sources such as grapes, and blueberries [8]. Previous studies have demonstrated that RES has anti-obesity and anti-inflammatory effects [9]. Previous animal studies have highlighted that RES can improve insulin signaling, decrease body weight, and lower fat accumulation [10]. RES primarily targets sirtuins (SIRTs), a family of nicotinamide adenine dinucleotide (NAD+)-dependent deacetylases localized in various cellular compartments [11]. SIRT1 is localized in the nucleus, whereas SIRT3 is the main deacetylase in the mitochondria [11,12]. Several studies have reported that both SIRT1 and SIRT3 are stimulated by calorie restriction and exercise [13,14]. Furthermore, RES activates SIRT1 directly [15] or rather indirectly by activation of the highly related metabolic sensor AMP-activated protein kinase (AMPK) [16]. In addition, animal studies have reported that RES modulates mitochondrial oxidative capacity and biogenesis via the activation of the AMPK–SIRT1–PGC-1α axis [13,14,15,16]. SIRT3 plays a pivotal role in regulating mitochondrial fatty acid and glucose oxidation. Multiple studies have shown that SIRT3 is downregulated in obesity and diabetes [17,18]. Treatment with RES improves insulin sensitivity and decreases body weight via the SIRT3-dependent pathway [17,18,19]. In the current study, we aimed to investigate whether the combination of a low-fat diet (LFD) and RES treatment improved the body weight, lipid profile, and insulin sensitivity in high fat-diet (HFD)-fed mice.

## 2. Methods

### 2.1. Animals

Mice received care and were treated according to the guidelines of Yarmouk university on animal care, and all procedures performed on mice were approved by the university’s animal welfare committee. BALB/c white albino male mice at age 8 weeks were fed either a high-fat (HF) diet (60% calories from fat, Research Diets Inc., New Brunswick, NJ 08901, USA) or a low-fat diet (12% calories from fat, PicoLab Laboratory Rodent Diet, 5l0D) for a 12-week period. Mice were randomly assigned into 1 of 4 groups: group 1: mice (*n* = 8) received only an LFD for the total period of 12 weeks, group 2: mice (*n* = 8) received an HFD only for the total period of 12 weeks, group 3: mice (*n* = 8) were fed with an HFD for 8 weeks to induce obesity and followed by intraperitoneal injection of RES (30 mg/Kg body wt.) every other day for 4 weeks (HFD + RES), or group 4: mice (*n* = 8) were fed with an HFD for 8 weeks to induce obesity and were then switched to an LFD and treated with RES for a further 4 weeks (HFD-LFD + RES). Skeletal muscles were then excised from pentobarbital euthanized mice, and immediately frozen to the temperature of liquid nitrogen.

### 2.2. Oral Glucose Tolerance Test

Intraperitoneal glucose tolerance test was performed following overnight fasting using 2 g/kg body weight [20]. Glucose levels were then assessed at times 0, 30, 60, and 120 min from tail vein blood samples using an Accu-Check^®^ Aviva glucometer.

### 2.3. Muscle Tissue Preparation for Metabolic Profiling

For triacylglyceride level assessment, approximately 10 mg of frozen gastrocnemius tissue was extracted with a 2:1 chloroform-methanol extraction method and quantified with an enzymatic assay kit, as previously described [21]. Diacylglycerol levels were assessed as previously described [22]. In brief, 5 mg of frozen gastrocnemius tissue was homogenized with 1 mM NaCl and then extracted with 1:2 chloroform/methanol and mixed. About 20 µL for each sample was measured by thin-layer chromatography assay. Ceramide content was also measured by thin-layer chromatography assay as described previously [22].

### 2.4. Quantitative Real-Time Reverse Transcriptase-PCR (qRT-PCR)

The extraction of total RNA from gastrocnemius muscles was performed using the TRIzol Reagent (Ambion, Inc., Austin, TX, USA) according to the manufacturer’s guidelines. Two micrograms of RNA were treated with DNase I (Promega, Madison, WI, USA), using a First Strand cDNA Synthesis kit with oligo-dT15 primers, as described before [23]. Quantitative real-time PCR for the expression of SIRT1, SIRT3, PGC1α, AMPK, LCAD, and β-HAD were carried out on a Line-Gene 9600 Real-Time PCR system (Bioer Technology, Bingjiang, China), at 95 °C for 3 min followed by 95 °C for 30 s and 60 °C for 30 s, using beta actin (β-actin) as non-regulated reference gene. SYBR-PCR reactions were performed using the SYBR Premix Ex TaqII PCR master mix (Takara, San Jose, CA, USA) in 20 μL final volume (10 μL SYBR green, 1 μL forward primer, 1 μL reverse primer, 6 μL nuclease free water and 2 μL cDNA). Beta actin was used as endogenous control to correct for potential variation in RNA loading and quantification.

### 2.5. Assessment of β-Hydroxylacyl CoA Dehydrogenase (β-HAD) Activity

About 100 mg of frozen muscle tissue was homogenized with a buffer contains 0.05 M Tris–HCl, 10% glycerol, 1 mM EDTA, 0.02% Brij-35, and 1 mM dithiothretol in the presence of phosphatases and proteases inhibitors. Homogenates were then centrifuged at 800 g for 10 min, and the supernatant lysates were pipetted and stored in a deep freezer. β-HAD activity was assayed on total muscle lysates prepared from frozen gastrocnemius tissues. About 10 µL of muscle lysates (10 µg protein) were pipetted into a 96-well plate. 160 µL of 50 mM imidazole (pH 7.4) and 20 µL of 1.5 mM NADH were added to each well. 10 µL of 2 mM acetoacetyl CoA was added to initiate the reaction and the absorbance at a 340 nM wavelength was followed for 5 min using a spectrophotometer kinetic plate reader.

### 2.6. Assessment of Long Chain Acyl CoA Dehydrogenase (LCAD) Activity

LCAD activity was assayed based on the method described by Lehman et al. [24]. In brief, 20 µL of total muscle lysate (20 µg protein) was added to potassium phosphate buffer containing 200 µM ferriceneum hexafluorophosphate (Fc^+^PF6^−^), N-ethylmaleimide (500 µM), and EDTA (0.1 µM) at pH 7.2. After initiating the reaction by the addition of palmitoyl CoA (50 µM), the absorbance at 300 nM wavelength was followed for 2 min using a spectrophotometer kinetic plate reader. 

## 3. Statistical Analysis

Data are represented as means ± S.E.M. Comparisons were performed by one-way ANOVA followed by Bonferroni’s multiple-comparison test whenever differences were detected. Differences were considered significant at *p* < 0.05.

## 4. Results

**Figure 1:**
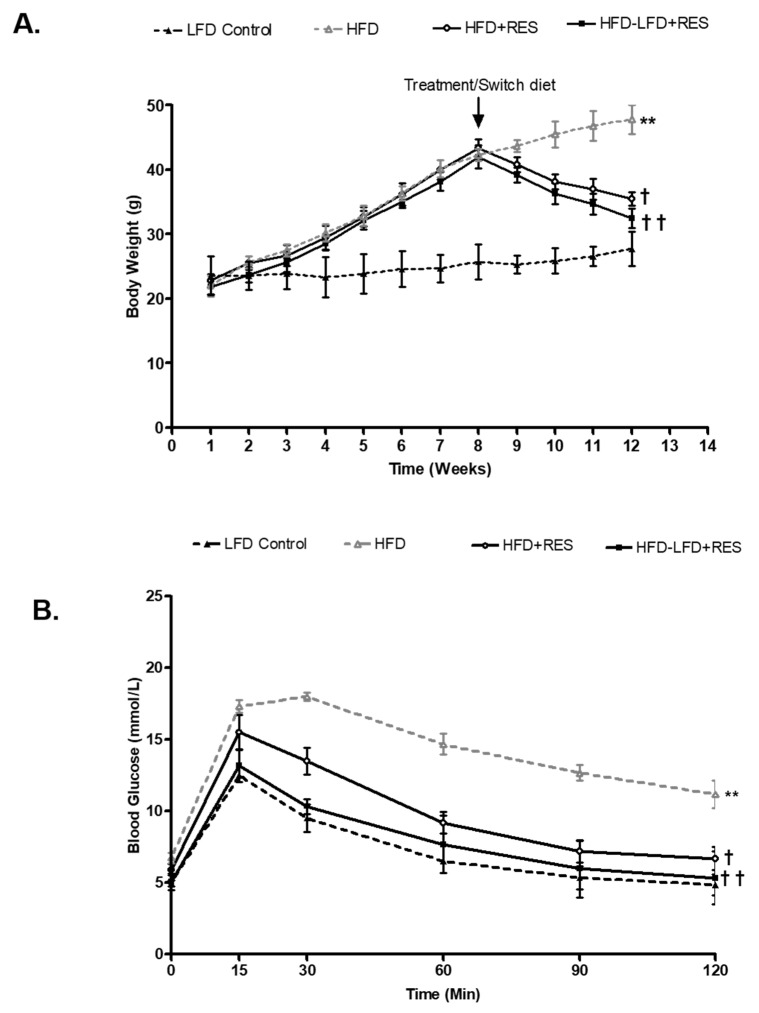
Effect of a low-fat diet and resveratrol treatment on weight gain and glucose tolerance in obese mice. (**A**): Body weight (g) over the 12-week study protocol in LFD control, HFD, HFD and RES, and HFD-LFD + RES groups. (**B**): Glucose tolerance at 12 weeks in LFD control, HFD, HFD and RES, and HFD-LFD + RES groups. ** *p* < 0.01 vs. LFD control; † *p* < 0.05; †† *p* < 0.01 vs. HFD. Values represents mean ± S.E.M. (*n* = 6–8).

Body weight was significantly elevated after eight weeks of HFD feeding in all groups compared to the baseline (Figure 1A). HFD-induced obese mice were subsequently divided into a group treated with RES only for a further 4 weeks (HFD + RES) or a group that was switched to LFD and treated with RES (HFD-LFD + RES) for four weeks. After 12 weeks, the HFD mice had significantly higher body weights compared to the LFD control group (23.71 ± 1.95 vs. 47.83 ± 2.27; *p* < 0.05).

### 4.1. Resveratrol Treatment Decreases Body Weight and Improves Glycemia in HFD-Induced Obese Mice

RES-treated mice showed a significant decrease in body weight compared to HFD-induced obese non-treated mice. Furthermore, the obese mice that were treated with RES and LFD for 4 weeks had more weight loss compared to the group treated with RES only (33.51 ± 1.05 vs. 27.50 ± 1.52; *p* < 0.05). Similarly, impaired glucose tolerance had developed in the HFD mice at 8 weeks compared to LFD group (Figure 1B). At 12 weeks, glucose tolerance was significantly improved in RES + LFD and RES-only groups compared to the obese HFD mice (71.3 ± 1.17 vs. 46.1 ± 1.82 and 40.9 ± 1.75, respectively; *p* < 0.05). 

### 4.2. Effects of Weight Loss on Gene Regulators of Fatty Acid and Glucose Metabolism

**Figure 2:**
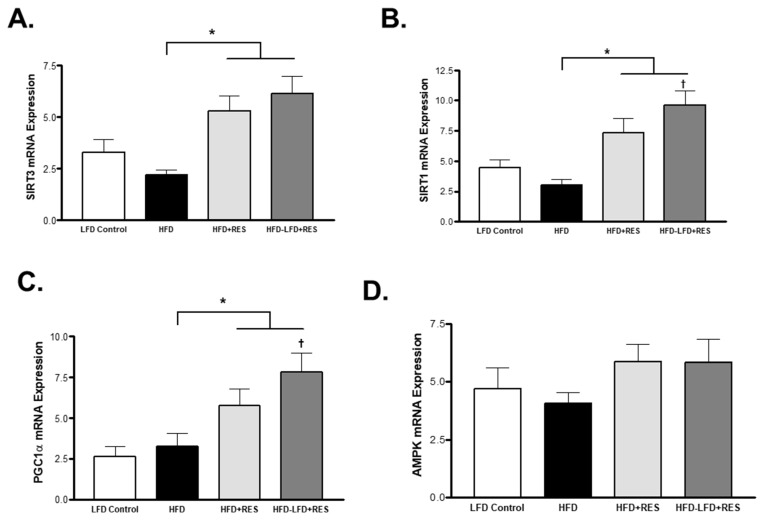
mRNA expression levels of sirtuins and oxidative capacity genes in Gastrocnemius muscle. (**A**): SIRT3 mRNA expression. (**B**): SIRT1 mRNA expression. (**C**): PGC1α mRNA expression. (**D**): AMPK mRNA expression. * *p* < 0.05 vs. HFD; † *p* < 0.05 vs. HFD + RES. Values represents mean ± S.E.M. (*n* = 6–8).

SIRT3 is localized in the mitochondria and is considered one of the main regulators of glucose and fatty acid oxidative metabolism. On the other hand, SIRT1 is localized in the nucleus and has important role in regulation of genes involved in metabolism. Therefore, we examined the gene expression of SIRT1 and SIRT3 in all groups. The SIRT3 level of mRNA expression was significantly increased by RES treatment alone (*p* < 0.05) and in the group that was treated by RES and switched to an LFD (*p* < 0.01) compared to the HFD group (Figure 2A). As such, SIRT1 expression was significantly higher in both groups that were treated with RES alone (HFD + RES) and the mice that were treated with RES and diet (HFD-LFD + RES) (Figure 2B). Moreover, there was a significant increase in the mRNA expression of SIRT1 in the HFD-LFD + RES mice compared to the mice treated with RES alone (Figure 2B). Peroxisome proliferator-activated receptor gamma coactivator 1-alpha (PGC1α) is a pivotal transcriptional regulator of genes and enzymes involved in fatty acid uptake and metabolism [25]. AMP-activated protein kinase (AMPK) is another metabolic sensor that can modulate the activity of PGC1α by phosphorylation. mRNA expression of PGC1α was significantly increased in the RES treated group compared to the HFD-induced obese non-treated mice (Figure 2C). Furthermore, PGC1α gene expression was significantly increased in the HFD-LFD + RES mice compared to the mice treated with RES alone (Figure 2C). Interestingly, no changes in the level of mRNA expression of AMPK were observed among the groups (Figure 2D). 

### 4.3. Resveratrol Treatment Increases the Activity of Fatty Acid Oxidation Enzymes

**Figure 3:**
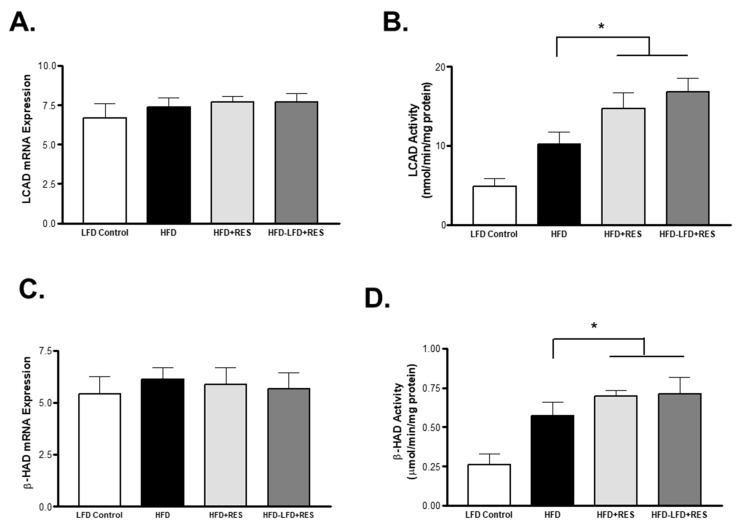
Effects of diet and resveratrol treatment on fatty acid oxidation enzymes. (**A**): LCAD mRNA expression. (**B**): LCAD enzyme activity. (**C**): β-HAD mRNA expression. (**D**): β-HAD enzyme activity. * *p* < 0.05 vs. HFD. Values represents mean ± S.E.M. (*n* = 6–8).

β-Hydroxyacyl CoA dehydrogenase (β-HAD) and long chain acyl CoA dehydrogenase (LCAD) are the main enzymes involved in the fatty acid β-oxidation pathway. We therefore assessed the level of gene expression of these enzymes in muscles from all groups. The levels of the LCAD gene were not changed with type of diet or treatment (Figure 3A). However, the levels of LCAD activity were significantly increased by RES treatment alone and in the group that was treated with RES and switched to an LFD (*p* < 0.05) compared to the HFD group (Figure 3B). The levels of β-HAD mRNA expression were also unaltered in the muscles of all groups (Figure 3C). However, a significant increase in β-HAD activity was observed in the RES-treated mice (Figure 3D).

### 4.4. Skeletal Muscles Lipid Metabolite Content in HFD-Induced Obese Mice after Treatment with Resveratrol

**Figure 4:**
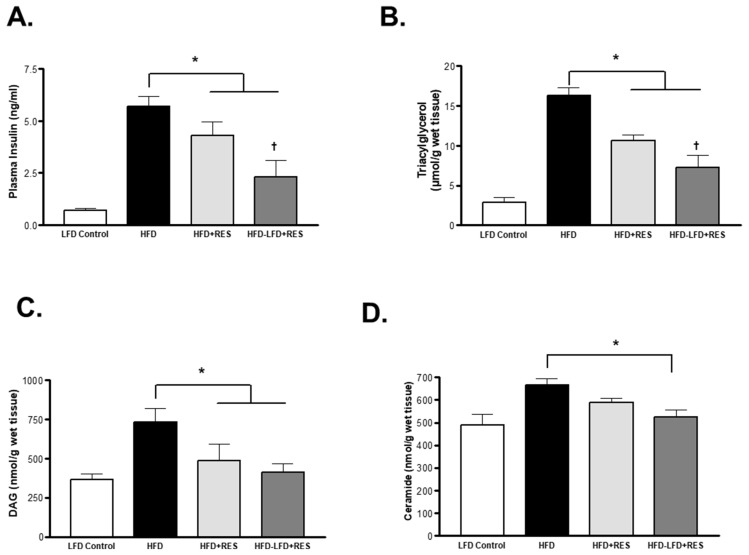
Muscle metabolic profile and plasma insulin levels in HFD treated mice. (**A**): Plasma insulin levels. (**B**): Gastrocnemius triacylglycerol content. (**C**): Diacylglycerol content. (**D**): Ceramide content. * *p* < 0.05 vs. HFD; † *p* < 0.05 vs. HFD + RES. Values represents mean ± S.E.M. (*n* = 6–8).

It is well established that obesity is associated with increased insulin secretion in humans and in obese animal models. We observed huge increases in plasma insulin levels in the HFD obese mice (Figure 4A). Interestingly, RES treatment significantly decreased the levels of plasma insulin levels in the HFD + RES mice (*p* < 0.05), and we noticed a further decrease in plasma insulin levels in the HFD-LFD + RES group (*p* < 0.01). On the other hand, the levels of lipid metabolites such as triacylglycerol (TAG) was significantly increased with HFD feeding (Figure 4B), and treatment with RES decreased the accumulation of TAG in the HFD + RES mice and significantly decreased the skeletal muscles TAG content in the HFD-LFD + RES group compared to the HFD + RES-treated mice (Figure 4B). Likewise, diacylglycerol (DAG) content was significantly lower in the HFD + RES and HFD-LFD + RES mice compared to the obese non-treated HFD mice (Figure 4C). In addition, we observed a significant decrease (*p* < 0.05) in ceramide muscle content in the HFD-LFD + RES mice only compared to the HFD obese mice (Figure 4C). Combined, this suggests that the increase in insulin secretion seen in the obese HFD mice was due to accumulation of lipid metabolites, and treatment with RES and diet enhanced lipid accumulation and insulin secretion.

## 5. Discussion

This study demonstrated several novel observations regarding the use of resveratrol and diet to treat obesity. First, weight loss due to a diet switch from an HFD to an LFD and RES treatment restored insulin sensitivity and improved overall glucose metabolism. Another desirable effect of weight loss was that the accumulation of lipid metabolite (e.g., TAG and DAG) was significantly reduced in the RES and diet combination group accompanied by a significant improvement in insulin secretion. Secondly, RES and diet treatment upregulated the expression of SIRT1, SIRT3, and PGC1α. However, it was interesting to note that AMPK expression levels were not affected by RES or diet treatment. Finally, the increase in PGC1α gene expression was accompanied by significant increase in the expression and activity of fatty acid oxidation enzymes LCAD and β-HAD in the group treated with LFD plus RES. Collectively, our data clearly demonstrated that there was a combined additive protective effect of RES and diet on weight loss, a decrease in lipid accumulation, and an improvement of overall insulin signaling. 

Previous studies have identified RES as a non-selective activator of SIRT1 [26,27,28]. In addition, SIRT1 is induced both by calorie restriction and exercise training [29,30]. In agreement, our results showed that SIRT1 levels of expression was increased by RES treatment or by diet. Interestingly, using RES and LFD simultaneously resulted in further increases in SIRT1 gene expression, as shown by our data. This suggest that there is a synergistic effect for RES and diet on the upregulation of SIRT1 levels. AMPK is another important molecular target for RES treatment [31]. However, there are disagreements about whether RES activates SIRT1 directly or indirectly by the activation of AMPK [16]. Park et al. suggested that the activation of AMPK increases NAD+ levels, which leads to the activation of SIRT1 [32]. We found that SIRT1 activation was dissociated from AMPK activity in the RES and diet-treated obese group, and our results suggest that the desirable metabolic effects of RES treatment were directly via the activation of SIRT1. Interestingly, a previous study showed that the effects of RES treatment were blunted on SIRT1 knockout mice [33]. Our data and others indicate that SIRT1 is required for the beneficial effects of RES treatment. PGC1α is a pivotal regulator of mitochondrial biogenesis and oxidative metabolism, and a downstream effector of the SIRT1-AMPK signaling pathway [34]. Previous human studies have suggested that PGC1α expression and activity is inhibited in obesity and type 2 diabetes [35,36], which leads to a disturbance in mitochondrial oxidative capacity and decreased insulin sensitivity. In a human study, calorie restriction for 6 months upregulated the expression of SIRT1 and PGC1α and improved insulin secretion [37]. In support, recent animal studies have shown improvements in mitochondrial biogenesis and oxidative capacity after treatment with various doses of RES on different animal models of obesity [38,39,40]. Together, these findings from animal and human studies indicate that resveratrol increases energy expenditure and mitochondrial biogenesis via the AMPK–SIRT1–PGC-1α axis. 

In this study, mice were injected intraperitoneally with RSV at a dose of 30 mg/kg/body weight as used in previous animal studies [28,41,42]. At this dose of RSV, we did not notice any side effects between the treatment groups. In a fatty liver mice model, treatment with RSV oral dose of 30 mg/kg body weight downregulated lipogenic genes and improved liver function [43]. Moreover, SIRT1 activation by intraperitoneal injection of RSV (25 mg/kg) alleviated cardiac dysfunction via improving mitochondrial function in diabetic cardiomyopathy mice [44]. It was previously reported that a daily dosage of 1 g up to 5 g of RSV is considered safe in humans [45]. In heart failure patients, an oral dose of 100 mg RSV daily given for 3 months significantly improved tissue perfusion and oxygen supply [46]. Moreover, in a double-blind, randomized, placebo-controlled clinical trial including 44 healthy subjects, 400 mg/daily of RSV for one month showed a protective effect against atherosclerosis in healthy individuals [47]. Furthermore, the long-term use of low doses of RSV (8 mg/daily/1 year) downregulates pro-inflammatory microRNAs and cytokines in type 2-hypertensive patients [48].

In mitochondria, SIRT3 plays a pivotal role in regulating the activity of fatty acid oxidation enzymes by deacetylation [49]. SIRT3 was induced by RES treatment, which increases fatty acid oxidation and ameliorates fat accumulation in HFD-induced obese mice [50]. Conflicting results have emerged regarding the role of deacetylation in controlling the activity of the major enzymes involved in the fatty acid oxidation pathway. In the liver, the deacetylation of LCAD by SIRT3 enhances fatty acid oxidation [51]. On the other hand, acetylation resulted in activation of β-HAD enzymes in muscle cells [52]. Moreover, SIRT3 expression and activity were inhibited in obese dam offspring concomitant with the hyperacetylation and inactivation of fatty acid oxidation enzyme LCAD [53]. Our study implicated that RES induced SIRT3, which leads to deacetylation and activation of the fatty acid oxidation enzymes LCAD and β-HAD. Previous studies have suggested that in obesity and type 2 diabetes, accumulation of lipid intermediates, such as triacylglycerol, diacylglycerol, and ceramide have a deleterious effect on skeletal muscles insulin sensitivity, thus increasing fatty acid oxidation in the muscle should decrease the accumulation of lipid intermediates and improve insulin secretion [25]. In agreement, our data showed that treatment with RES or diet enhanced muscle fatty acid oxidation and protect against insulin resistance by reducing the accumulation of these lipid metabolites.

## 6. Conclusions

**Figure 5:**
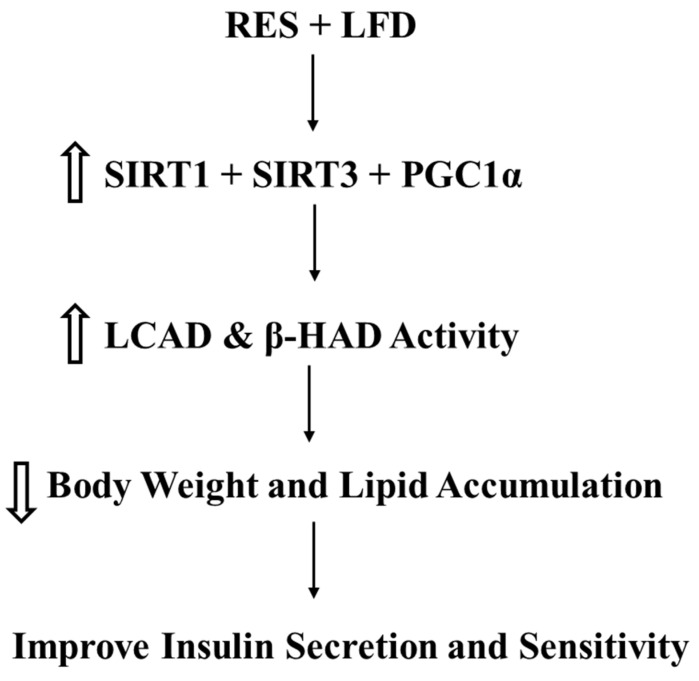
Model for the mechanism of action of resveratrol and diet on glucose and lipid metabolism.

In conclusion, a decrease in SIRT1 and SIRT3 seen following chronic HFD feeding lead to lipid accumulation and impaired insulin signaling. A combination treatment with RES and diet significantly improved insulin signaling, decreased lipid accumulation, and enhanced fatty acid oxidation (Figure 5). Our results offer new insight on using both RES and diet as a novel treatment for obesity and type 2 diabetes.

## Data Availability

Not applicable.
